# A mutation that blocks integrin α_4_β_7_ activation prevents adaptive immune-mediated colitis without increasing susceptibility to innate colitis

**DOI:** 10.1186/s12915-020-00784-6

**Published:** 2020-06-10

**Authors:** Hailong Zhang, Yajuan Zheng, Youdong Pan, Changdong Lin, Shihui Wang, Zhanjun Yan, Ling Lu, Gaoxiang Ge, Jinsong Li, Yi Arial Zeng, Jianfeng Chen

**Affiliations:** 1 State Key Laboratory of Cell Biology, Shanghai Institute of Biochemistry and Cell Biology, Center for Excellence in Molecular Cell Science, Chinese Academy of Sciences; University of Chinese Academy of Sciences, 320 YueYang Road, Shanghai, 200031 China; 2 Department of Orthopedics, the First People’s Hospital of Wujiang District, 169 GongYuan Road, Suzhou, 215200 China; 3 School of Life Science, Hangzhou Institute for Advanced Study, University of Chinese Academy of Sciences, Hangzhou, 310024 China

**Keywords:** Inflammatory bowel disease, Integrin α_4_β_7_, Activation, Lymphocyte

## Abstract

**Background:**

β_7_ integrins are responsible for the efficient recruitment of lymphocytes from the blood and their retention in gut-associated lymphoid tissues. Integrin α_4_β_7_ binds MAdCAM-1, mediating rolling adhesion of lymphocytes on blood vessel walls when inactive and firm adhesion when activated, thereby controlling two critical steps of lymphocyte homing to the gut. By contrast, integrin α_E_β_7_ mediates the adhesion of lymphocytes to gut epithelial cells by interacting with E-cadherin. Integrin β_7_ blocking antibodies have shown efficacy in clinical management of inflammatory bowel disease (IBD); however, fully blocking β_7_ function leads to the depletion of colonic regulatory T (Treg) cells and exacerbates dextran sulfate sodium (DSS)-induced colitis by evoking aberrant innate immunity, implying its potential adverse effect for IBD management. Thus, a better therapeutic strategy targeting integrin β_7_ is required to avoid this adverse effect.

**Results:**

Herein, we inhibited integrin α_4_β_7_ activation in vivo by creating mice that carry in their integrin β_7_ gene a mutation (F185A) which from structural studies is known to lock α_4_β_7_ in its resting state. Lymphocytes from β_7_-F185A knock-in (KI) mice expressed α_4_β_7_ integrins that could not be activated by chemokines and showed significantly impaired homing to the gut. The β_7_-F185A mutation did not inhibit α_E_β_7_ activation, but led to the depletion of α_E_β_7_^+^ lymphocytes in the spleen and a significantly reduced population of α_E_β_7_^+^ lymphocytes in the gut of KI mice. β_7_-F185A KI mice were resistant to T cell transfer-induced chronic colitis, but did not show an increased susceptibility to DSS-induced innate colitis, the adverse effect of fully blocking β_7_ function.

**Conclusions:**

Our findings demonstrate that specific inhibition of integrin α_4_β_7_ activation is a potentially better strategy than fully blocking α_4_β_7_ function for IBD treatment.

## Background

Inflammatory bowel disease (IBD), including ulcerative colitis (UC) and Crohn’s disease (CD), is an idiopathic intestinal disorder caused by an inappropriate inflammatory response to intestinal microbes in a genetically susceptible host [1, 2]. The pronounced infiltration of both innate and adaptive immune cells is observed in the gut of UC and CD patients [3, 4], suggesting that both types of immunity are involved in the progression of IBD [5]. The migration of leukocytes into inflamed intestinal tissue is tightly regulated by specific cell adhesion molecules [6]. The cell-surface glycoprotein β_7_ integrins regulate the homing and retention of lymphocytes in the gut-associated lymphoid tissues (GALT) [7]. Integrin α_4_β_7_ mediates the homing of lymphocytes into the GALT via interaction with mucosal addressin cell adhesion molecule-1 (MAdCAM-1) on the intestinal vasculature [8, 9], whereas integrin α_E_β_7_ facilitates the retention of lymphocytes in the gut epithelium through binding to E-cadherin [10]. The highest expression of α_4_β_7_ can be found on memory gut-homing CD4^+^ T cells, and it is also expressed on other T cell subsets (T_H_2 and T_H_17) and B cells as well as some other leucocytes [11–13]. α_E_β_7_ mainly expresses in CD8^+^ intraepithelial T cells, T_H_9, CD69^+^α_E_^+^ intestinal tissue-resident memory T (T_RM_) cells, Treg cells, and mucosal dendritic cell subsets [14–16]. Of note, integrin β_7_ has emerged as a promising drug target for the treatment of IBD since blocking β_7_ function suppresses the migration of inflammatory lymphocytes to the GALT [17, 18] and consequently inhibits adaptive immune-mediated colitis [19].

Clinical studies have reported that two humanized monoclonal antibodies that can block integrin β_7_ function, vedolizumab (anti-α_4_β_7_ antibody) and etrolizumab (anti-β_7_ antibody), effectively maintain clinical remission in IBD patients [20, 21]. Vedolizumab specifically blocks α_4_β_7_ interaction with MAdCAM-1 by binding to the specificity-determining loop (SDL) that projects from the β_7_ I domain at the interface with the α_4_ β-propeller domain [22]. This epitope is only accessible in the heterodimer of α_4_ and β_7_ subunits. Etrolizumab binds to the β_7_ subunit of both α_4_β_7_ and α_E_β_7_ integrins and blocks the interaction of β_7_ integrins with their ligands MAdCAM-1 and E-cadherin, respectively [23]. Interestingly, UC patients who received 100 mg etrolizumab show a significantly higher remission rate than patients receiving 300 mg etrolizumab [21]. Moreover, aggravated colitis is observed in a small percentage of UC patients treated with a high dose of vedolizumab [20]. These reports suggest that excessive inhibition of β_7_ function may have adverse effects on the management of IBD under certain conditions. Indeed, total loss of α_4_β_7_ function has been shown to lead to the depletion of colonic Treg cells and consequently exacerbate dextran sulfate sodium (DSS)-induced colitis by evoking aberrant innate immunity [24]. Moreover, vedolizumab treatment significantly suppresses homing of Treg cells to the gut in UC patients [25]. Therefore, a more specific approach to suppress adaptive immune response without evoking aberrant innate immunity in colitis could be more efficacious for IBD treatment.

Integrin α_4_β_7_ mediates rolling and firm adhesion of lymphocytes when inactive and activated, respectively [22, 26], controlling two critical steps in tissue-specific homing of lymphocytes [27, 28]. Integrin activation can be dynamically regulated via a cluster of three metal ion-binding sites in the β I domain [26, 29]. A cation-π interaction in the human β_7_ I domain between F185 in the SDL and the synergistic metal ion-binding site (SyMBS) cation has been shown to be essential for the activation of α_4_β_7_ [30]. Disruption of the cation-π interaction through mutation of F185 to Ala inhibits α_4_β_7_ activation and α_4_β_7_-mediated cell migration.

Herein, we generated knock-in (KI) mice bearing the integrin β_7_-F185A mutation which blocked α_4_β_7_ activation. Moreover, this mutation did not inhibit the chemokine-induced activation of α_E_β_7_, but induced the significant reduction of α_E_β_7_ expression in KI mice. Similar to lymphocytes from integrin β_7_ knock-out (KO) mice, lymphocytes from KI mice showed significantly deficient homing to the GALT, and CD4^+^CD45RB^high^ T cells from KI mice could not induce colitis in a T cell transfer model. Notably, KI mice exhibited sufficient colonic Treg cells, in stark contrast to the depletion of colonic Treg cells in β_7_ KO mice, and thus avoided the aberrant innate immunity in DSS-induced colitis. Taken together, specific blockade of integrin α_4_β_7_ activation is sufficient to prevent adaptive immune-mediated colitis without increasing susceptibility to innate colitis and therefore is a potentially better treatment for IBD than complete blockade of α_4_β_7_ function.

## Results

### Generation of KI mice bearing the β_7_-F185A mutation

**Fig. 1:**
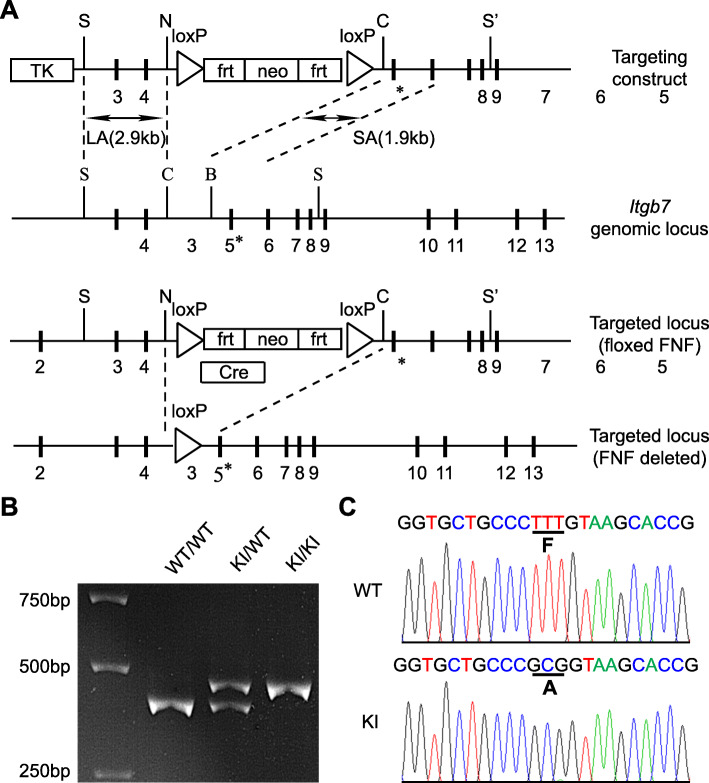
Generation of β_7_-F185A KI mice. **a** Targeted insertion to the *Itgb7* locus of the floxed *neo* cassette, and the mutated exon 5 (5*) that contains β_7_-F185A. The targeting vector, the WT *Itgb7* locus, the targeted *Itgb7* allele containing floxed *neo* cassette, and the mutated *Itgb7* (F185A) allele are shown. Exons are shown as thick lines, as well as long arm (LA) and short arm (SA) of homology are also shown. The *loxP*-flanked *neo* cassette is deleted by intercrossing the mutant mouse strains with an EIIa-Cre strain, leaving 1 loxP site. S, SacII; N, NotI; C, ClaI; S’, SaII. **b** Genotyping and confirmation of deleted *neo* cassette by PCR. Genomic DNA isolated from tails was used for PCR analyses. PCR bands are shown for WT (WT/WT, 360 bp), heterozygote (KI/WT, 380 and 360 bp), and homozygote (KI/KI, 380 bp) samples. **c** Sequencing analysis of WT and KI mice. DNA sequencing confirmed a phenylalanine-to-alanine substitution at position 185 of the mouse β_7_ integrin gene in KI mice

To block the activation of integrin α_4_β_7_ in vivo, we generated a mouse with the F185A point mutation in the mouse β_7_ gene, thereby disrupting the synergistic metal-binding site (SyMBS) cation-F185 interaction within β_7_ I domain and locking α_4_β_7_ in a resting state, as shown previously for human β_7_ [30]. Specifically, using a replacement-type gene-targeting strategy [31], a phenylalanine-to-alanine mutation (F185A) was introduced into the mouse integrin β_7_ gene, *Itgb7*. Embryonic stem cells were electroporated with the targeting construct encoding mutant β_7_-F185A and were then injected into blastocysts. Chimeric mice with germline transmission of the targeted allele were crossed with an EIIa-cre mouse to excise the *neo* cassette (Fig. 1a). The correct integration of the mutant β_7_ gene *Itgb7*^F185A^ was confirmed by PCR and DNA sequencing (Fig. 1b, c). *Itgb7*^F185A/F185A^ (β_7_-F185A KI) mice showed no abnormalities by visual inspection and had a normal life span. In addition to the β_7_-F185A KI mice, we also included β_7_ KO (*Itgb7*^−/−^) mice in all experiments to compare the effects of blockade of α_4_β_7_ activation with total loss of β_7_ function.

### Reduced lymphocytes in the gut of β_7_-F185A KI mice

**Fig. 2:**
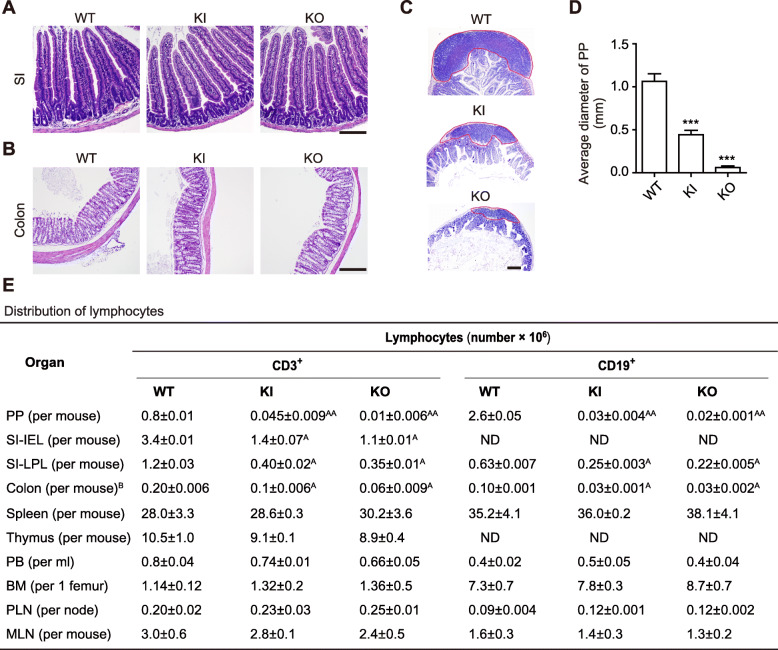
Reduced lymphocytes in the GALT of β_7_-F185A KI mice. Representative histological sections of the small intestine (SI) (**a**), colon (**b**), and Peyer’s patch (PP) (**c**) of WT, β_7_-F185A KI (KI), and β_7_-KO (KO) mice were analyzed by hematoxylin and eosin staining. Scale bars, 100 μm. **d** Quantification of the average diameter of PP in the individual group of mice (*n* = 9 per group). ***P < 0.001 (Student’s *t* test). **e** Flow cytometry enumeration of lymphocyte distribution in lymphoid organs from the individual group of mice (*n* = 9 per group). PB, peripheral blood; PLN, peripheral lymph node; MLN, mesenteric lymph node; BM, bone marrow; IEL, intraepithelial lymphocyte; LPL, lamina propria lymphocyte. Results are presented as cell number × 10^6^, ^A^*P* < 0.01; ^AA^*P* < 0.005 (Student’s *t* test). ^B^The cecum was excluded. ND, not detected. Data are mean ± s.d. of at least 3 independent experiments (**d**, **e**)

The small intestine (SI) and colon of KI and KO mice exhibited basically normal architectures (Fig. 2a, b); however, Peyer’s patches (PP) with decreased cellularity and rudimentary follicles were observed in KI and KO mice compared with wild-type (WT) mice (Fig. 2c, d). The spleen (SP), peripheral lymph nodes (PLN), and mesenteric lymph nodes (MLN) were indistinguishable among WT, KI, and KO mice (Additional file 1: Figure S1). We next analyzed the distribution of lymphocytes in the lymphoid organs of these mice. Flow cytometric analyses showed that compared with WT mice, KI mice contained significantly fewer lymphocytes in the gut including fewer intraepithelial lymphocyte (IEL) and lamina propria lymphocyte (LPL) in the SI and fewer T and B cells in the PP and colon (Fig. 2e). Moreover, KO mice showed a greater decrease in CD3^+^ T cells in the gut than did KI mice. Thus, both integrin β_7_-F185A mutation and β_7_ KO can specifically inhibit lymphocyte recruitment to the GALT. It is noteworthy that β_7_ KO results in a greater inhibition of T cell recruitment to the gut.

### Chemokine fails to promote α_4_β_7_-mediated adhesion of β_7_-F185A KI lymphocytes

**Fig. 3:**
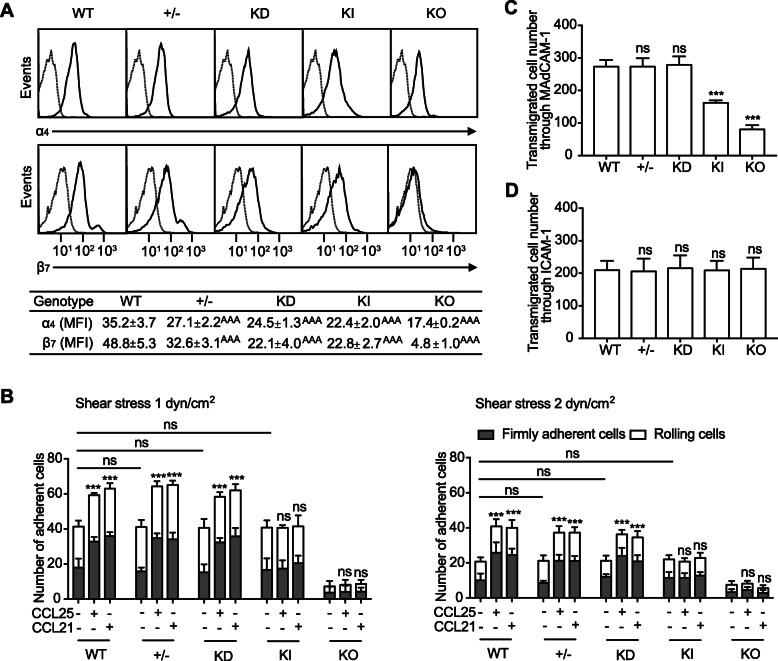
Impaired adhesion and transmigration of β_7_-F185A KI lymphocytes. **a** Cell surface expression of integrins ⍺_4_ and β_7_ on splenic lymphocytes from WT, *Itgb7*^*+/−*^ (+/−), β_7_ knock-down (KD), KI, and KO mice. All viable lymphocytes were gated using a combination of forward angle and side scatter to exclude dead cells and debris. And the results were presented as histograms for ⍺_4_ and β_7_ expression. The numbers within the table show the specific mean fluorescence intensities of FIB504 (anti-β_7_) and GK1.5 (anti-α_4_) mAbs. **b** Adhesion of WT, +/−, KD, KI, and KO splenic lymphocytes to MAdCAM-1 at 1 dyn/cm^2^ or 2 dyn/cm^2^ before and after chemokine stimulation. **c**, **d** Transmigration of WT, +/−, KD, KI, and KO splenic lymphocytes toward a serum gradient through MAdCAM-1-coated (**c**) or ICAM-1-coated (**d**) permeable insert was examined using a modified Boyden chamber assay with a transwell tissue culture system. ***P < 0.001; ns, not significant. ^AAA^*P* < 0.001 (Student’s *t* test in **a**–**d**). Data are mean ± s.d. of at least 3 independent experiments (**a**–**d**). The asterisk in **b** indicates the changes of total adherent cells

We found that splenic lymphocytes from KI mice showed an approximately 50% reduction in β_7_ integrin cell surface expression compared with cells from WT mice (Fig. 3a). Decreased expression of α_4_ integrin was also observed in KI and KO mice, likely resulting from the reduction in β_7_ expression (Fig. 3a). Although quantitative reverse transcription polymerase chain reaction (qRT-PCR) showed that β_7_ mRNA level was comparable between WT and KI splenic lymphocytes (Additional file 1: Figure S2A), flow cytometric analysis of permeabilized cells indicated that the total expression of β_7_ integrin, including cell surface and intracellular expression, was decreased in KI lymphocytes (Additional file 1: Figure S2B).

Next, we examined α_4_β_7_-mediated splenic lymphocyte adhesion on MAdCAM-1 substrates using a parallel wall flow chamber. Considering the reduced expression of α_4_β_7_ in KI lymphocytes, we included the *Itgb7*^+/−^ (+/−) and β_7_ knock-down (KD) splenic lymphocytes as controls because those cells also express reduced levels of β_7_ integrins (Fig. 3a). In the presence of the physiologic cations 1 mM Ca^2+^/Mg^2+^, which maintain α_4_β_7_ predominantly in the resting state [26, 30], WT, +/−, KD and KI lymphocytes showed similar adhesive behavior on MAdCAM-1 substrates in flow (Fig. 3b), indicating that the F185A mutation and the reduction in α_4_β_7_ expression have no effect on the adhesion of unstimulated lymphocytes to MAdCAM-1. As expected, β_7_ KO lymphocytes rarely adhered to MAdCAM-1 due to the loss of α_4_β_7_ expression (Fig. 3b).

Activation of α_4_β_7_ by chemokines promotes the firm adhesion of lymphocytes to MAdCAM-1, which is a critical step during lymphocyte homing to the gut [32, 33]. The flow chamber results showed that chemokine (C-C motif) ligand 25 (CCL25) and chemokine (C-C motif) ligand 21 (CCL21) significantly increased the number of firmly adherent WT, +/−, and KD splenic lymphocytes on MAdCAM-1 substrates, whereas these chemokines were not able to increase the adhesion of KI cells to MAdCAM-1 (Fig. 3b), indicating that chemokines cannot activate the α_4_β_7_-F185A mutant, thus failing to promote α_4_β_7_-mediated cell adhesion. Despite the reduced α_4_β_7_ expression on +/− and KD splenic lymphocytes, these cells showed adhesive behavior similar to WT cells upon chemokine treatment, suggesting that the impaired adhesion of β_7_-F185A cells is not due to the reduction in α_4_β_7_ expression.

### β_7_-F185A KI lymphocytes show impaired α_4_β_7_-mediated transmigration

Next, we studied the impact of defective β_7_ integrin activation on α_4_β_7_-mediated lymphocyte transmigration using a transwell assay. Compared with WT lymphocytes, significantly fewer KI cells transmigrated through the MAdCAM-1-coated insert (Fig. 3c), indicating that blockade of β_7_ integrin activation perturbed α_4_β_7_-mediated cell transmigration. Moreover, knockout of β_7_ led to a further decrease in lymphocyte transmigration across MAdCAM-1 substrates (Fig. 3c). Similar numbers of WT, +/−, and KD splenic lymphocytes transmigrated through a MAdCAM-1-coated insert, suggesting that the decreased expression of β_7_ integrin does not affect α_4_β_7_-mediated cell transmigration. As a control, WT, +/−, KD, KI, and KO splenic lymphocytes showed the intact ability of β_2_ integrin to mediate cell transmigration through an ICAM-1-coated insert (Fig. 3d).

To confirm the effects of β_7_-F185A mutation on the adhesion and migration of human lymphocytes, we established Jurkat T-β_7_ WT and Jurkat T-β_7_ F185A, which stably expressed similar level of WT or F185A β_7_ (Additional file 1: Figure S3A). Because Jurkat T cells lack CCL25 chemokine receptor 9 (CCR9), CCR9 was also co-transfected into those cells. Consistent with the results of mouse lymphocytes, CCL25 and CCL21 only promoted the adhesion of Jurkat T-β_7_ WT but not Jurkat T-β_7_ F185A to MAdCAM-1 (Additional file 1: Figure S3B). Compared with Jurkat T-β_7_ WT cells, significantly fewer Jurkat T-β_7_ F185A cells transmigrated through the MAdCAM-1-coated insert (Additional file 1: Figure S3C). Taken together, inhibition of integrin β_7_ activation has the same effects on the adhesion and migration of mouse and human lymphocytes.

### ⍺_E_β_7_^+^ lymphocytes are reduced in β_7_-F185A KI mice

In addition to integrin ⍺_4_β_7_, the β_7_ subunit also forms a heterodimer with integrin ⍺_E_ subunit. The α_E_β_7_ integrin facilitates the retention of lymphocytes in the gut epithelial layer via interactions with E-cadherin. In contrast to the inhibition of ⍺_4_β_7_ activation by β_7_-F185A, CCL21 and CCL25 promoted the adhesion of both Jurkat T-α_E_β_7_ WT and Jurkat T-α_E_β_7_ F185A to E-cadherin (Additional file 1: Figure S4), indicating that β_7_-F185A mutation does not inhibit chemokine-induced activation of α_E_β_7_. Unlike integrin α_4_β_7_ which binds to ligand via the metal ion-dependent adhesion site (MIDAS) in β_7_ subunit, α_E_β_7_ has an I domain in the α_E_ subunit and binds to E-cadherin through MIDAS in α_E_ I domain [26, 34]. Thus it is not surprising that the β7-F185A mutation inhibits the cytokine-activated β_7_-mediated adhesion to  MAdCAM-1, but not the α_E_-mediated adhesion to E-cadherin. We observed, however, a significant decrease of ⍺_E_β_7_^+^ splenic lymphocytes in KI mice and the depletion of ⍺_E_β_7_^+^ splenic lymphocytes in KO mice (Additional file 1: Figure S5A). Furthermore, flow cytometry showed that the percentages of ⍺_E_^+^ IEL and ⍺_E_^+^ LPL significantly decreased in the gut of KI mice and almost disappeared in KO mice (Additional file 1: Figure S5B), these percentage changes being seen in the context of the reduced total IEL and LPL populations in these mice (Fig. 2e). In addition, the expression level of α_E_ is significantly decreased in ⍺_E_^+^ IEL and LPL populations in KI and KO mice (Additional file 1: Figure S5C). Because ⍺_E_β_7_ is critical for the retention of intestinal lymphocytes and the α_E_ KO has been shown to induce the reduction of intestinal lymphocytes in mice [35], it is tempting to speculate that β_7_-F185A mutation-induced downregulation of ⍺_E_β_7_ expression impaired the retention of intestinal lymphocytes in KI mice. To summarize, although the β_7_-F185A mutation did not inhibit the chemokine-induced activation of α_E_β_7_, it led to the significant reduction of α_E_β_7_-expressing lymphocytes and reduced the α_E_ expression level in ⍺_E_β_7_^+^ IELs and LPLs in KI mice, which would be expected to impair lymphocyte retention in the gut.

### Suppressed β_7_-F185A lymphocyte homing to the GALT

**Fig. 4:**
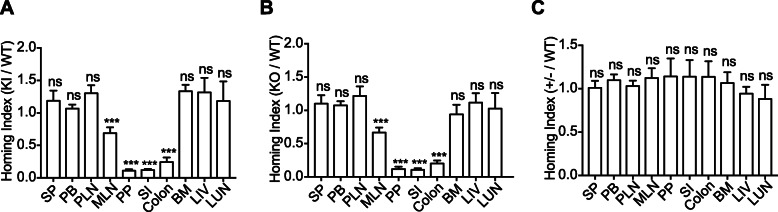
Reduced in vivo homing of β_7_-F185A lymphocytes to the GALT. In vivo competitive homing of splenic lymphocytes from WT mice and KI (**a**) or KO (**b**) or +/− mice (**c**). The ratio of KI, KO, or +/− lymphocytes over WT lymphocytes that homed to specific tissues was determined 18 h after injection. SP, spleen; PB, peripheral blood; PLN, peripheral lymph node; MLN, mesenteric lymph node; PP, Peyer’s patch; SI, small intestine; BM, bone marrow; LIV, liver; LUN, lung. ***P < 0.001; ns, not significant (Student’s *t* test). Data are mean ± s.d. of at least 3 independent experiments

Next, we investigated whether lymphocyte homing to the GALT was affected by the defective activation of β_7_ integrin using a competitive homing assay [36]. Splenic lymphocytes freshly isolated from WT and KI mice were fluorescently labeled with CellTrace Violet and CellTrace Yellow, respectively. Equal numbers of WT and KI lymphocytes were mixed and then intravenously administered into C57BL/6J recipient mice. Organs were harvested 18 h after administration, and the homing indices were then determined. WT and KI lymphocytes homed equally well to the SP, PLN, bone marrow (BM), liver (LIV), and lung (LUN), whereas homing of KI lymphocytes to MLN, PP, SI, and the colon was severely decreased in comparison (Fig. 4a). This indicates that blockade of β_7_ integrin activation specifically suppresses lymphocyte homing to the GALT. In addition, KO lymphocytes showed decreased homing to the GALT similar to that seen with KI lymphocytes (Fig. 4b), while the homing of +/− lymphocytes was not affected (Fig. 4c). Collectively, these results demonstrate that either integrin β_7_-F185A mutation or β_7_ KO can efficiently inhibit gut-specific homing of lymphocytes.

### Reduced capacity of β_7_-F185A T cells to induce chronic colitis

**Fig. 5:**
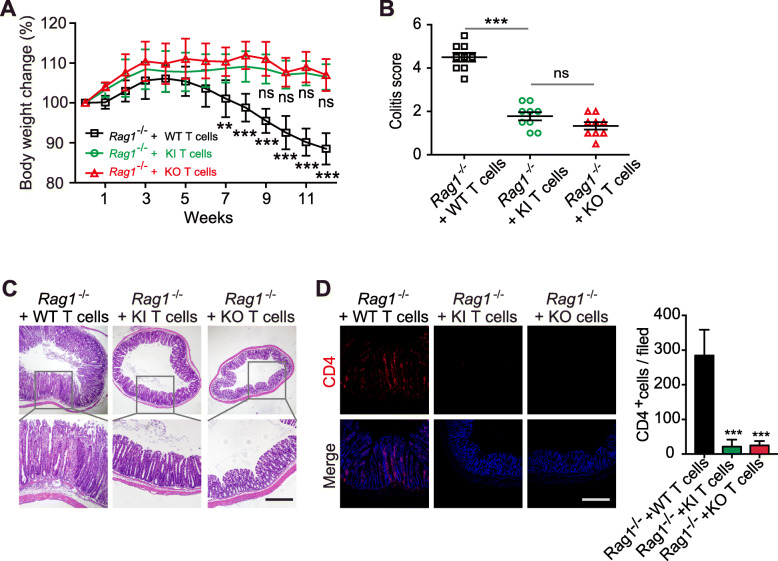
Reduced capacity of β_7_-F185A T cells to induce chronic colitis. *Rag1*^−/−^ mice (*n* = 9 per group) were given 1 × 10^5^ CD4^+^CD45RB^high^ T cells isolated from WT, KI, or KO splenic lymphocytes and monitored for 12 weeks. **a** Body weight change after T cell transfer. **b** Quantitative histopathologic grading of colitis severity. **c** Hematoxylin and eosin staining of colon sections at week 12 after T cell transfer. **d** Immunofluorescence staining and CD4^+^ T cell quantification of colon sections with anti-CD4 (red) and DAPI (blue) as in **d**. Scale bars, 100 μm (**c**, **d**). **P < 0.005, ***P < 0.001; ns, not significant (two-way ANOVA in **a**, Student’s *t* test in **b**). Data are mean ± s.d. of at least 3 independent experiments (**a**, **b**)

Integrin β_7_-mediated T lymphocyte recruitment to the gut has been implicated in the pathogenesis of chronic colitis [19, 20]. Therefore, we studied the capacity of β_7_-F185A T cells to induce intestinal inflammation using a T cell transfer model of chronic colitis [37]. As previously described [37], *Rag1*^−/−^ mice transferred with 1 × 10^5^ WT CD4^+^CD45RB^high^ T cells presented progressive loss of body weight and higher colitis activity score (Fig. 5a, b) and showed clinical symptoms of severe colitis including massive infiltration of mononuclear cells in colonic lamina propria, disruption of epithelial boundaries, and disappearance of goblet cells (Fig. 5c). On the contrary, *Rag1*^−/−^ mice reconstituted with the same number of KI or KO CD4^+^CD45RB^high^ T cells appeared healthy, showed no progressive body weight loss (Fig. 5a, b), and maintained normal colonic mucosal architecture (Fig. 5c). Consistent with the above results, markedly fewer KI and KO CD4^+^ T cells were present in the colonic lamina propria compared with WT CD4^+^ T cells (Fig. 5d). Thus, blocking α_4_β_7_ activation is equivalent to fully inhibiting α_4_β_7_ function with regard to suppressing the recruitment of inflammatory T cell to the gut and subsequently eliminating adaptive immune-mediated chronic colitis.

### β_7_-F185A KI mice are resistant to DSS-induced acute colitis

**Fig. 6:**
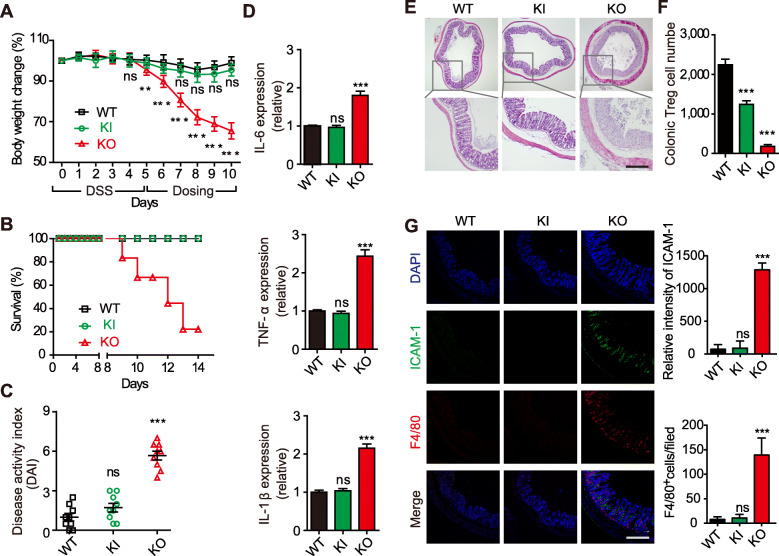
β_7_-F185A KI mice are resistant to DSS-induced acute colitis. Eight-week-old WT, KI, and KO mice were treated with low concentration of DSS (2%) for 5 days, followed by regular drinking water (dosing). **a**–**c** Body weight change (**a**), survival ratio (**b**), and disease activity index (**c**) were obtained from individual groups of mice (*n* = 6 per group). **d** Quantitative PCR analysis of IL-6, TNF-α, and IL-1β expression in distal colon tissue from individual groups of mice (*n* = 6 per group) at day 10 after DSS treatment. Results are normalized to *GAPDH*. **e** Hematoxylin and eosin staining of distal colon sections at day 10 after DSS treatment. **f** Flow cytometric analysis of Treg cell number in colonic tissues from individual groups of mice (*n* = 6 per group) at day 4 after DSS treatment. **g** Immunofluorescence staining and quantification of F4/80^+^ macrophages and ICAM-1 expression in distal colon sections with anti-ICAM-1 (green), anti-F4/80 (red), and DAPI (blue) at day 4 after DSS treatment. Scale bars, 100 μm (**e**, **g**). **P < 0.005; ***P < 0.001; ns, not significant (two-way ANOVA in **a**, Student’s *t* test in **c**, **d**, **f**, and **g**). Data are mean ± s.d. of at least 3 independent experiments (**a**, **c**, **d**, **f**, **g**)

In addition to adaptive immunity, innate immunity also contributes to the pathogenesis of IBD [38]. In both IBD patients and the DSS-induced colitis model, macrophages were considered a critical factor involved in disease progression [39, 40]. Moreover, total loss of α_4_β_7_ function has been shown to lead to colonic Treg depletion, which promotes ICAM-1 expression and macrophage infiltration into the colon and exacerbates DSS-induced colitis [24]. Therefore, we next evaluated the effect of blocking α_4_β_7_ activation on the susceptibility to innate immune-mediated acute colitis using the DSS colitis model [41]. Instead of using high concentration of DSS (3.5–5%) to induce severe acute colitis [42, 43], mice were treated with low concentration of DSS (2%) to induce impairment of mucosal barrier function in the gut and moderate acute colitis [24, 39, 44]. Compared with the rapid body weight loss of DSS-treated β_7_ KO mice, DSS-treated WT and KI mice showed similar mild body weight changes throughout the observation period (Fig. 6a). Notably, WT and KI mice exhibited a 100% survival rate at day 14 after initial DSS treatment compared with the 20% survival rate of KO mice (Fig. 6b). In contrast to the severe clinical symptoms of colitis found in KO mice, WT and KI mice developed more mild clinical symptoms of colitis, including lower colitis activity score (Fig. 6c), decreased colonic expression of proinflammatory cytokines (IL-6, TNF-α, and IL-1β) (Fig. 6d), fewer inflammatory infiltrates, and moderate disruption of mucosal structures (Fig. 6e). Of note, KI mice retained around 55% of Treg cells in the colonic lamina propria compared with the approximately 92% depletion of colonic Treg cells in KO mice (Fig. 6f). In contrast to the increased ICAM-1 expression and macrophage infiltration in the colon of KO mice, KI mice exhibited no visible ICAM-1 expression or only mild macrophage infiltration into the colon (Fig. 6g). Because the colonic Treg cells have been shown to inhibit ICAM-1 expression and macrophage infiltration in the colon in DSS colitis [24], it is tempting to speculate the retained colonic Treg cells in KI mice may be related to the susceptibility to DSS-induced innate colitis.

Taken together, specific blockade of integrin α_4_β_7_ activation is sufficient to prevent adaptive immune-mediated colitis without increasing susceptibility to innate colitis, suggesting the efficacy of this approach in treating IBD. Of note, specifically blocking α_4_β_7_ activation retains colonic Treg cells, thus appears to be advantageous over fully blocking α_4_β_7_ function by avoiding aberrant innate immune response. Therefore, the targeting of integrin α_4_β_7_ activation for the treatment of IBD has the potential to induce fewer adverse effects than complete blockade of α_4_β_7_ function.

## Discussion

Integrin α_4_β_7_ mediates rolling adhesion and firm adhesion of lymphocytes pre- and post-activation respectively [22, 26], controlling two critical steps of lymphocyte homing to the gut [27, 28]. Blocking the function of α_4_β_7_ efficiently suppresses the recruitment of inflammatory lymphocytes to the gut and consequently inhibits adaptive immune-mediated colitis [19, 45]. The US Food and Drug Administration has approved the integrin α_4_β_7_ blocking antibody vedolizumab for the treatment of adults with moderately to severely active UC or CD. Clinical studies report that vedolizumab effectively maintains clinical remission in some IBD patients; however, aggravated colitis is observed in a small percentage of UC patients treated with high dose of vedolizumab [20]. One possible reason could be that the excessive loss of α_4_β_7_ function leads to colonic Treg depletion, which exacerbates colitis by evoking aberrant innate immune response in a DSS colitis model [24]. Indeed, a study has reported that vedolizumab treatment significantly suppresses Treg cells homing to the gut in UC patients [25]. Instead of completely blocking the function of integrin, an appropriate modulation of integrin function might be required not only to suppress aberrant immune response-induced inflammation but also to maintain immune homeostasis [46, 47]. In this study, we demonstrate that specific inhibition of integrin α_4_β_7_ activation is sufficient to prevent adaptive immune-mediated colitis without increasing susceptibility to innate colitis, suggesting a potentially better treatment for IBD than complete blockade of α_4_β_7_ function. It provides novel mechanism of action for the development of new IBD drugs.

Leukocyte recruitment to the gut is dynamically regulated by adhesion cascades including cell capture, slow rolling, adhesion strengthening, intravascular crawling, and transcellular transmigration [48]. Previous in vitro studies have shown that resting α_4_β_7_ mediates robust cell rolling on immobilized MAdCAM-1 substrates and firm cell adhesion upon activation [26, 30]. Full blockade of α_4_β_7_ function disables the ability of this integrin to mediate both rolling and firm cell adhesion, whereas specifically blocking α_4_β_7_ activation only partially inhibits α_4_β_7_-mediated cell adhesion, which still allows this integrin to support cell rolling [30]. Consistent with this, our in vitro data showed that there were significantly more β_7_-F185A KI cells than β_7_ KO cells that adhered to (mostly rolling events) and transmigrated through MAdCAM-1 substrates (Fig. 3b, c). Interestingly, the in vivo short-term competitive homing assay showed that there was no significant difference between the suppression of recruitment of KI and KO lymphocytes to the GALT (Fig. 4), which is consistent with the data that CD4^+^CD45RB^high^ T cells from KI and KO mice are similarly impaired in their ability to induce colitis in a T cell transfer model. Thus, in a manner similar to full inhibition of α_4_β_7_ function, blocking α_4_β_7_-mediated firm cell adhesion through specific inhibition of α_4_β_7_ activation suppresses lymphocyte homing into the gut. This indicates both approaches are capable of inhibiting adaptive immune-mediated colitis.

As shown by clinical studies that high doses of etrolizumab reduce the remission rate of IBD patients, it is possible that homing of Treg cells is reduced due to the excessive inhibition of β_7_ function, as is the KO phenotype. It is noteworthy that β_7_ KO mice did show a greater decrease in CD3^+^ T cells in the gut compared with β_7_-F185A KI mice (Fig. 2e). Importantly, there were significantly more colonic Treg cells in β_7_-F185A KI mice than in KO mice after DSS treatment, suggesting the α_4_β_7_-mediated rolling adhesion may contribute to the maintenance of the colonic Treg cell population. Although the number of colonic Treg cells in β_7_-F185A KI mice was less than that in WT mice, there appear to be enough colonic Treg cells present to be resistant to the aberrant innate immune response that exacerbates gut inflammation in the DSS colitis model (Fig. 6). Thus, specifically blocking α_4_β_7_ activation avoids excessive depletion of colonic Treg cells, which is important for suppressing aberrant innate immune responses under certain conditions.

In addition to integrin α_4_β_7_, the β_7_ subunit also forms a heterodimer with the α_E_ subunit. Through an interaction with E-cadherin, α_E_β_7_ mediates cell-to-cell interactions between T cells and intestinal epithelial cells, thereby playing an important role in the retention of lymphocytes in mucosal tissue [49]. Integrin α_E_ KO mice display a reduction in the number of lymphocytes residing in the intestinal epithelium and lamina propria [35]. The reduced expression of integrin α_E_ mainly impairs the retention of lymphocytes in the gut, but has little effect on the gut homing of lymphocytes [50, 51]. In this study, we did observe significantly reduced expression of α_E_β_7_ in KI lymphocytes, the depletion of the α_E_β_7_^+^ lymphocytes in the spleen, and significantly reduced the population of α_E_β_7_^+^ IEL and LPL in the gut of KI mice. β_7_-F185A mutation did not inhibit the chemokine-induced activation of α_E_β_7_, but downregulated the expression of α_E_β_7_ in lymphocytes, thereby impairing lymphocyte retention in the gut.

It is also noteworthy that a small population of β_7_-high splenic lymphocytes disappeared in KI mice (Fig. 3a). Further analysis showed that this group of cells mainly consisted of CD8^+^ T cells (79.05%), CD4^+^ T cells (8.74%), CD4^+^CD25^+^ Treg cells (1.42%), and CD19^+^ B cells (3.90%) (Additional file 2: Table S1). Whether the depletion of this β_7_-high lymphocyte population is related to colitis needs to be further studied.

Etrolizumab is an anti-β_7_ monoclonal antibody that is able to inhibit the functions of both α_4_β_7_ and α_E_β_7_ integrins, whereas vedolizumab is an anti-α_4_β_7_ antibody which only inhibits the function of α_4_β_7_. It is reported that vedolizumab is more effective in UC than in CD [52]. One potential explanation is that UC is related to T_H_2 and T_H_17 cells, which mainly express α_4_β_7_. By contrast, etrolizumab is more effective in inflammatory environments rich in CD69^+^α_E_^+^ intestinal T_RM_ cells and T_H_9 cells because these cells express high level of α_E_β_7_ [14, 15]. In addition, a phase II study reports that patients with elevated intestinal expression of α_E_β_7_ may have an increased chance of clinical remission in response to etrolizumab treatment [53]. Thus, a personalized selection of vedolizumab or etrolizumab for the treatment of different IBD patients might benefit the clinical remission. It is noteworthy that blocking β_7_ activation leads to the loss of α_E_β_7_ expression in lymphocytes as shown in this study, which should be able to suppress the functions of both α_E_β_7_ and α_4_β_7_ integrins.

## Conclusions

Collectively, β_7_-F185A mutation not only inhibited integrin α_4_β_7_ activation thus reducing the α_4_β_7_-mediated homing of lymphocytes into the gut, but also induced the downregulation of integrin α_E_β_7_ expression in lymphocytes and impaired the retention of lymphocytes in the gut. Importantly, rather than completely inhibiting β_7_ function, inhibiting the activation of α_4_β_7_ could retain enough Treg cells in the gut, thus could prevent adaptive immune-mediated colitis without increasing the susceptibility to innate colitis, suggesting this is potentially a better strategy than fully blocking β_7_ function for IBD treatment. Therefore, the development of antibodies and small molecule antagonists that specifically block α_4_β_7_ activation may provide a new approach for clinical management of IBD.

## Methods

### Generation of β_7_-F185A KI mice

For the construction of *Itgb7* gene-targeting vector, a 2.9-kb SacII/NotI fragment containing exon 3, 4 and a 1.3-kb ClaI/SaII fragment containing exon 5, 6 were subcloned into the upstream regions and downstream of the *loxP*-flanked *neo* gene, respectively. F185A was introduced in exon 5 by site-directed mutagenesis. The exonic sequences in the targeting vector were confirmed by DNA sequencing. The targeting vector was transfected into ES cells of the 129/SvJ mouse strain. G418-resistant ES cell clones were screened for homologous recombination. The correctly targeted ES clones were injected into C57BL/6J blastocysts, and the resulting chimeric males were mated to C57BL/6J females for germline transmission. The *neo* cassette was deleted after crossed with EIIa-cre mice. The offspring were screened for heterozygosity by PCR (Primers, Additional file 2: Table S2) to confirm in vivo deletion of the *neo* cassette. The mice that were backcrossed into the C57BL/6J background for ten generations were used in all experiments.

### Mice

*Itgb7*^−/−^ mice, *Rag1*^−/−^ mice, and EIIa-cre mice with C57BL/6J background were from Jackson Laboratory (Bar Harbor, ME). All mice were maintained under specific pathogen-free conditions. Age- and sex-matched mice were used at 8–10 weeks of age. All animal studies were approved by the Institutional Animal Care and Use Committee of the CAS Center for Excellence in Molecular Cell Science, Shanghai Institute of Biochemistry and Cell Biology, Chinese Academy of Sciences.

### Antibodies and reagents

Monoclonal antibodies (mAbs) specific for mouse CD3 (17A2), CD4 (GK1.5), CD8 (53-6.7), CD19 (1D3), CD45RB (C303.16A), and integrin α_4_ (9C10) were all from BD Bioscience (San Jose, CA); mAb to mouse CD3 (17A2) was from Invitrogen (Carlsbad, CA); mAb to human/mouse E-Cadherin (24E10) was from Cell Signaling; mAbs to mouse F4/80 (BM8), CD25 (PC61.5), and Foxp3 (FJK-16s) were from eBioscience (San Diego, CA); mAb to mouse ICAM-1 (M-19) was from Santa Cruz (Santa Cruz, CA); mAb to integrin β_7_ (N1N3) was from GeneTex (Irvine, CA); rat mAb FIB504 against human/mouse β_7_ and rat mAb M290 against human/mouse ⍺_E_ were prepared by using hybridomas (Developmental Studies Hybridoma Bank, University of Iowa); mAbs to mouse CD19 (1D3), human CCR9 (L053E8), FITC goat anti-rat IgG, and FITC goat anti-mouse IgG were from BioLegend; 4,6-diamidino-2-phenylindole (DAPI) was from Sigma (St. Louis, MO); CellTrace Violet Cell Proliferation Kit and CellTrace Yellow Cell Proliferation Kit were from Thermo Fisher Scientific (Waltham, MA). Human and mouse chemokines CCL21 and CCL25 were purchased from R&D Systems (Minneapolis, MN). Recombinant mouse extracellular maturation peptide E-cadherin (Asp157-Val709) fused with Fc tag were expressed in 293T cells and purified by protein A (Pierce) affinity chromatography.

### Cell lines

Jurkat T cell line expressing WT or mutant human ⍺_4_β_7_ was generated by infection with the recombinant lentivirus, and human CCR9 was infected at the same time. Similarly, Jurkat T cell line expressing WT or mutant mouse ⍺_E_β_7_ was generated by infection with the recombinant lentivirus. Integrins and CCR9 expressing efficiency were assessed by flow cytometry 72 h after transfection using a FACS Celesta flow cytometer (BD Biosciences).

### Mononuclear cell isolation and flow cytometry

Lymphocyte cells were isolated from the spleen, thymus, PB, BM, PLN, MLN, PP, and colon as previously described [25, 54–56]. Briefly, lymphocytes were isolated from the spleen, thymus, PLN, MLN, and PP by dispersion through a fine wire mesh. To harvest the IEL and LPL, the intestinal tube was opened laterally and cut into pieces measuring 0.5 cm. Intestinal pieces were incubated for 3 or 5 consecutive times in HBSS/EDTA (1 mmol/L DTT) for 20 min at 37 °C with stirring. For the isolation of LPL, tissue fragments were further subjected to 2 rounds of treatment in RPMI-1640 with 5% FBS, collagenase D, DNase I, and dispase for 30 min at 37 °C. The resulting suspensions were layered on a discontinuous 40%/70% Percoll gradient, centrifuged at 650*g* for 20 min, and IEL and LPL were recovered from the interphase. Aliquots of 1 × 10^6^ viable cells were stained with 50 μl of optimally titrated antibody cocktail in PBS containing 5% FBS. For intracellular staining of Foxp3, cells were fixed and permeabilized using the mouse Treg cell staining kit (eBioscience) before staining with anti-Foxp3 mAb. Cells were maintained on ice throughout the staining procedure. Samples were analyzed by using the FACS Celesta flow cytometer (BD Biosciences). All viable lymphocytes were gated using a combination of forward angle and side scatter to exclude dead cells and debris. Acquisition and analysis were performed on FlowJo (LLC, Ashland, Oregon, USA).

### Histology and immunofluorescence microscopy

Formalin-fixed, paraffin-embedded small intestine and distal colon sections of 4-μm thickness were mounted on glass slides and followed by hematoxylin and eosin staining. For immunostaining analysis, distal colons were collected at individual days after the initial DSS treatment or T cell transfer. Cryostat sections were made permeable with cold acetone and blocked with 1% (wt/vol) BSA. Samples were incubated with fluorochrome-conjugated anti-F4/80 (5 μg/ml), anti-ICAM-1 (10 μg/ml), or anti-CD4 (5 μg/ml), and counterstaining of nuclei was with DAPI (1 μg/ml). Images were acquired with a Leica TCS SP8 confocal microscope (Leica, Mannheim, Germany).

### RNA isolation and real-time quantitative PCR

Total RNA was extracted from mouse splenic lymphocytes or distal colonic tissues with TRIzol reagent according to the manufacturer’s instructions (Invitrogen, Carlsbad, CA). For cDNA synthesis, RNA was reverse-transcribed with an M-MLV reverse transcriptase (Promega, Madison, WI). Then, cDNA was amplified by real-time PCR (primers, Additional file 2: Table S3) with a SYBR Premix ExTaq kit (TaKaRa, Otsu, Japan) on an AbiPrism 7500 sequence detector (Applied Biosystems, Foster City, CA). The expression of target genes was normalized to the expression of the housekeeping gene GAPDH.

### Silencing of β_7_ in splenic lymphocytes

Silencing of β_7_ expression in WT mouse splenic lymphocytes was achieved by shRNA. Cells with β_7_ were generated by infection with the recombinant lentivirus, which harbors packaging plasmid psPAX2 (7.5 μg), envelope plasmid pMD2.G (3 μg), and transfer plasmid pLKO.1-shItgb7 (10 μg). The oligonucleotide targeting sequence for mouse β_7_ was 5′-CCCGTCTTCTAGTGTTCACTT-3′. Knockdown of β_7_ was confirmed by flow cytometry 72 h after transfection using a FACS Celesta flow cytometer (BD Biosciences).

### Flow chamber assay

Cell adhesion under physiologic shear stress was studied as previously described [26]. Briefly, a polystyrene petri dish was coated with 20 μl of MAdCAM-1/Fc (10 μg/ml) alone or with chemokines (2 μg/ml) in coating buffer (PBS, 10 mM NaHCO_3_, pH 9.0) for 1 h at 37 °C followed by blocking with 2% BSA in coating buffer for 1 h at 37 °C. Cells were diluted to 1 × 10^6^ cells/ml in HBSS (10 mM HEPES, 1 mM Ca^2+^/Mg^2+^) and immediately infused in the flow chamber using a syringe pump through the flow chamber at a constant flow of 1 dyn/cm^2^ or 2 dyn/cm^2^. All adhesive interactions between the flowing cells and the coated substrates were determined by manually tracking the motions of individual cells as previously described [57]. The motion of each adherent cell was monitored for 10 s following the initial adhesion point, and two categories of cell adhesion were defined. Adhesion was defined as rolling adhesion if the adherent cells were followed by rolling motions ≥ 5 s with a velocity of at least 1 μm/s for splenic lymphocytes or 2 μm/s for Jurkat T cells due to the larger cell size, whereas a firmly adherent cell was defined as a cell that remained adherent and stationary for at least 10 s. For integrin ⍺_E_β_7_-mediated adhesion, a polystyrene petri dish was coated with 20 μl of mouse E-cadherin-Fc (40 μg/ml) alone or with chemokines (2 μg/ml) in coating buffer. Cells were diluted to 1 × 10^6^ cells/ml in HBSS and immediately infused in the flow chamber using a syringe pump through the flow chamber at a constant flow of 1 dyn/cm^2^. Then, cells were allowed to adhere for 2 min, and the number of adherent cells was determined.

### Transwell migration assay

Transwell migration was performed using Millicell inserts with 5-μm pore size for splenic lymphocytes and 8-μm pore size for Jurkat T cells (Millipore, Billerica, MA). Ten micrograms per milliliter of MAdCAM-1/Fc or 10 μg/ml ICAM-1/Fc was coated on the upper surface of inserts, and the lower chamber was filled with 1 ml RPMI 1640 medium with 10% FBS. 2 × 10^5^ cells in 0.2-ml serum-free RPMI 1640 medium were added into the upper chamber. Cells were incubated for 6 h at 37 °C in 5% CO_2_. Cells remaining on the upper surface of the inserts were scraped with a cotton swab, and cells migrating to the bottom surface were counted after fixation with 3.7% formaldehyde and staining with DAPI.

### Competitive in vivo homing assay

A competitive homing assay was conducted as previously described [36]. 2 × 10^7^ splenic lymphocytes from heterozygote (+/−), KI, or KO mice labeled with CellTrace Yellow were mixed with the same number of WT cells labeled with CellTrace Violet and injected intravenously into C57BL/6J mice. An aliquot was saved to assess the input ratio (calculated as [Yellow^+^][13, 15]_input_/[Violet^+^]_input_). Recipient mice were scarified 18 h after injection, and lymphocytes from tissues were harvested. The homing index was calculated as the [Yellow^+^]_tissue_/[Violet^+^]_tissue_ ratio to the input ratio.

### T cell transfer colitis

CD4^+^ CD45RB^high^ naive T cells were isolated from mouse splenic lymphocytes using LSRII (BD Bioscience) cell sorting with FITC-conjugated anti-CD45RB and PE-conjugated anti-CD4 mAbs. 1 × 10^5^ WT, KI, or KO CD4^+^CD45RB^high^ cells in 0.2 ml PBS were injected intravenously into *Rag1*^−/−^ recipient mice, respectively. Mice were weighted weekly and observed for signs of illness as reported previously [58]. At week 12, mice were scarified, and histological tissue was taken and analyzed.

### DSS-induced acute colitis

Acute colitis was induced by the provision of 2% (wt/vol) DSS with molecular mass of 36–50 kDa (MP Biomedicals, Irvine, CA) in drinking water for a total of 5 days (days 0–5), followed by regulatory drinking water (days 6–15). Mice were assessed daily for body weight, diarrhea, and bloody stool. The disease activity index (DAI) and histological damage were assessed by trained individuals blinded to the treatment groups, as reported previously [58, 59]. DAI was calculated as the combined score of stool consistency (0, normal; 1, moist/sticky; 2, soft; 3, diarrhea), presence of blood in the stool (0, no blood; 1, blood in the stool or around the anus; 2, for severe bleeding), and mouse appearance (0, normal; 1, ruffled fur or altered gait; 2, lethargic or moribund). The severity of colitis was assessed on stained colonic sections in a blinded fashion using established criteria based on crypt damage and ulceration. Crypt damage was scored as follows: 0, intact crypts; 1, loss of the basal one-third; 2, loss of the basal two-thirds; 3, entire crypt loss; 4, change of epithelial surface with erosion; and 5, confluent erosion. Ulceration was scored as follows: 0, absence of ulcer; 1, one or two foci of ulceration; 2, two to four foci of ulcerations; and 3, confluent or extensive ulceration. On day 10, mice were sacrificed and colons removed and analyzed. For interim analyses, experiments were done as described above but terminated on day 4. Colon tissues were used for histological and quantitative PCR analysis.

### Statistical analysis

Statistical analyses were performed with GraphPad PRISM software 5.0 (GraphPad Software, La Jolla, CA). Significances were determined by two-tailed Student’s *t* test or two-way ANOVA as indicated. *P < 0.01, **P < 0.005, and ***P < 0.001 were considered statistically significant in all figures.

## Supplementary information

**Additional file 1: Figure S1.** The secondary lymphoid tissues other than GALT appear normal in β7-F185A KI and β7-KO mice. Representative histological sections of the peripheral lymph node (PLN), mesenteric lymph node (MLN) and spleen (SP) of WT, β7-F185A KI (KI) and β7-KO (KO) mice were analyzed by hematoxylin and eosin staining. Scale bars, 500μm. **Figure S2.** Expression of integrins β7 in splenic lymphocytes of WT and β7-F185A mice. (**A**) Quantitative PCR analysis of integrin β**7** expression in splenic lymphocytes from WT and KI mice. Results are normalized to *GAPDH*. (**B**) Total (cell surface plus intracellular) protein expression of integrin β**7** in splenic lymphocytes was determined by flow cytometry using permeabilized cells. Data are mean ± s.d. of at least 3 independent experiments (**A-B**). **Figure S3.** Impaired adhesion and transmigration of Jurkat T-β7 F185A cells. (**A**) Expession of β7 and CCR9 in Jurkat T-β7 WT and Jurkat T-β7 F185A cell lines were determined by flow cytometry. (**B**) Adhesion of Jurkat T-β7 WT, Jurkat T-β7 F185A and Jurkat T cells to MAdCAM-1 substrates at 1 dyn/cm^2^ or 2 dyn/cm^2^ before and after chemokine stimulation. (**C**) Transmigration of Jurkat T-β7 WT, Jurkat T-F185A and Jurkat T cells through MAdCAM-1-coated insert. Data are mean ± s.d. of at least 3 independent experiments (**B C**). *** *P* < 0.001; ns, not significant (Student’s *t*-test). Asterisk in **B** indicates the changes of total adherent cells. **Figure S4.** β7-F185A mutation does not affect αEβ7-mediated cell adhesion to E-cadherin substrates. (**A**) Expession of β7 and αE in Jurkat T-αEβ7 WT and Jurkat T-αEβ7 F185A cell lines were determined by flow cytometry. The numbers within the panels show the specific mean fluorescence intensities of FIB504 (anti-β7) and M290 (anti-αE) mAbs. (**B**) Adhesion of Jurkat T-αEβ7 WT, Jurkat T-αEβ7 F185A and Jurkat T cells to the immobilized E-cadherin substrates (40 μg/ml) at 1dyn/cm^2^ before and after chemokine stimulation. αEβ7-E-cadherin binding was inhibited by pre-treatment of cells with 10 μg/ml αE blocking antibody M290. Data are mean ± s.d. of at least 3 independent experiments (**A-B**).*** *P* < 0.001; ns, not significant (Student’s *t*-test). **Figure S5.** Integrin αE^+^ lymphocytes in spleen, SI and colon. (**A**) Expression of αE in WT, +/-, KI and KO splenic lymphocytes was determined by flow cytometry. (**B**-**C**) Expression of αE in intestinal IEL and LPL was detected by flow cytometry. The numbers within the panels (**A-B**) show the percentage of αE^+^ lymphocytes. The numbers within the table (**C**) show the specific mean fluorescence intensities of M290 (anti-αE) mAb in αE^+^ lymphocytes. Data are mean ± s.d. of at least 3 independent experiments (**A**-**C**). ^AA^*P* < 0.05; ^AAA^*P* < 0.001 (Student's *t*-test in **C**). SP, spleen; SI, small intestine; IEL, intraepithelial lymphocyte; LPL, lamina propria lymphocyte.

**Additional file 2: Table S1.** The analysis of integrin β7-high splenic lymphocytes subsets in WT mice. **Table S2.** The list of primers used for genotyping. **Table S3.** The list of primers used for real-time quantitative PCR analyses

## Data Availability

All data generated or analyzed during this study are included in this published article and its additional information files.

## References

[1] Abraham C, Cho JH (2009). Inflammatory bowel disease. N Engl J Med.

[2] Fiskerstrand T, Arshad N, Haukanes BI, Tronstad RR, Pham KD, Johansson S, Havik B, Tonder SL, Levy SE, Brackman D (2012). Familial diarrhea syndrome caused by an activating GUCY2C mutation. N Engl J Med.

[3] Matsuoka K, Inoue N, Sato T, Okamoto S, Hisamatsu T, Kishi Y, Sakuraba A, Hitotsumatsu O, Ogata H, Koganei K (2004). T-bet upregulation and subsequent interleukin 12 stimulation are essential for induction of Th1 mediated immunopathology in Crohn’s disease. Gut.

[4] Franchimont D, Vermeire S, El Housni H, Pierik M, Van Steen K, Gustot T, Quertinmont E, Abramowicz M, Van Gossum A, Deviere J (2004). Deficient host-bacteria interactions in inflammatory bowel disease? The toll-like receptor (TLR)-4 Asp299gly polymorphism is associated with Crohn's disease and ulcerative colitis. Gut.

[5] Elson CO, Cong Y, McCracken VJ, Dimmitt RA, Lorenz RG, Weaver CT (2005). Experimental models of inflammatory bowel disease reveal innate, adaptive, and regulatory mechanisms of host dialogue with the microbiota. Immunol Rev.

[6] Rose DM, Alon R, Ginsberg MH (2007). Integrin modulation and signaling in leukocyte adhesion and migration. Immunol Rev.

[7] Wagner N, Lohler J, Kunkel EJ, Ley K, Leung E, Krissansen G, Rajewsky K, Muller W (1996). Critical role for beta7 integrins in formation of the gut-associated lymphoid tissue. Nature.

[8] Erle DJ, Briskin MJ, Butcher EC, Garcia-Pardo A, Lazarovits AI, Tidswell M (1994). Expression and function of the MAdCAM-1 receptor, integrin alpha 4 beta 7, on human leukocytes. J Immunol.

[9] Arihiro S, Ohtani H, Suzuki M, Murata M, Ejima C, Oki M, Kinouchi Y, Fukushima K, Sasaki I, Nakamura S (2002). Differential expression of mucosal addressin cell adhesion molecule-1 (MAdCAM-1) in ulcerative colitis and Crohn's disease. Pathol Int.

[10] Cepek KL, Shaw SK, Parker CM, Russell GJ, Morrow JS, Rimm DL, Brenner MB (1994). Adhesion between epithelial cells and T lymphocytes mediated by E-cadherin and the alpha E beta 7 integrin. Nature.

[11] Soler D, Chapman T, Yang LL, Wyant T, Egan R, Fedyk ER (2009). The binding specificity and selective antagonism of vedolizumab, an anti-alpha4beta7 integrin therapeutic antibody in development for inflammatory bowel diseases. J Pharmacol Exp Ther.

[12] Schweighoffer T, Tanaka Y, Tidswell M, Erle DJ, Horgan KJ, Luce GE, Lazarovits AI, Buck D, Shaw S (1993). Selective expression of integrin alpha 4 beta 7 on a subset of human CD4+ memory T cells with hallmarks of gut-trophism. J Immunol.

[13] Zeissig S, Rosati E, Dowds CM, Aden K, Bethge J, Schulte B, Pan WH, Mishra N, Zuhayra M, Marx M (2019). Vedolizumab is associated with changes in innate rather than adaptive immunity in patients with inflammatory bowel disease. Gut.

[14] Zundler S, Becker E, Spocinska M, Slawik M, Parga-Vidal L, Stark R, Wiendl M, Atreya R, Rath T, Leppkes M (2019). Hobit- and Blimp-1-driven CD4(+) tissue-resident memory T cells control chronic intestinal inflammation. Nat Immunol.

[15] Zundler S, Schillinger D, Fischer A, Atreya R, Lopez-Posadas R, Watson A, Neufert C, Atreya I, Neurath MF (2017). Blockade of alphaEbeta7 integrin suppresses accumulation of CD8(+) and Th9 lymphocytes from patients with IBD in the inflamed gut in vivo. Gut.

[16] del Rio ML, Rodriguez-Barbosa JI, Kremmer E, Forster R (2007). CD103- and CD103+ bronchial lymph node dendritic cells are specialized in presenting and cross-presenting innocuous antigen to CD4+ and CD8+ T cells. J Immunol.

[17] Gorfu G, Rivera-Nieves J, Hoang S, Abbott DW, Arbenz-Smith K, Azar DW, Pizarro TT, Cominelli F, McDuffie M, Ley K (2010). Beta7 integrin deficiency suppresses B cell homing and attenuates chronic ileitis in SAMP1/YitFc mice. J Immunol.

[18] Agace WW (2008). T-cell recruitment to the intestinal mucosa. Trends Immunol.

[19] Picarella D, Hurlbut P, Rottman J, Shi X, Butcher E, Ringler DJ (1997). Monoclonal antibodies specific for beta 7 integrin and mucosal addressin cell adhesion molecule-1 (MAdCAM-1) reduce inflammation in the colon of scid mice reconstituted with CD45RBhigh CD4+ T cells. J Immunol.

[20] Feagan BG, Greenberg GR, Wild G, Fedorak RN, Pare P, McDonald JW, Dube R, Cohen A, Steinhart AH, Landau S (2005). Treatment of ulcerative colitis with a humanized antibody to the alpha4beta7 integrin. N Engl J Med.

[21] Vermeire S, O'Byrne S, Keir M, Williams M, Lu TT, Mansfield JC, Lamb CA, Feagan BG, Panes J, Salas A (2014). Etrolizumab as induction therapy for ulcerative colitis: a randomised, controlled, phase 2 trial. Lancet.

[22] Yu Y, Zhu J, Mi LZ, Walz T, Sun H, Chen J, Springer TA (2012). Structural specializations of alpha (4) beta (7), an integrin that mediates rolling adhesion. J Cell Biol.

[23] Tang MT, Keir ME, Erickson R, Stefanich EG, Fuh FK, Ramirez-Montagut T, McBride JM, Danilenko DM (2018). Review article: nonclinical and clinical pharmacology, pharmacokinetics and pharmacodynamics of etrolizumab, an anti-beta7 integrin therapy for inflammatory bowel disease. Aliment Pharmacol Ther.

[24] Zhang HL, Zheng YJ, Pan YD, Xie C, Sun H, Zhang YH, Yuan MY, Song BL, Chen JF (2015). Regulatory T-cell depletion in the gut caused by integrin beta7 deficiency exacerbates DSS colitis by evoking aberrant innate immunity. Mucosal Immunol.

[25] Fischer A, Zundler S, Atreya R, Rath T, Voskens C, Hirschmann S, Lopez-Posadas R, Watson A, Becker C, Schuler G (2016). Differential effects of alpha 4 beta 7 and GPR15 on homing of effector and regulatory T cells from patients with UC to the inflamed gut in vivo. Gut.

[26] Chen J, Salas A, Springer TA (2003). Bistable regulation of integrin adhesiveness by a bipolar metal ion cluster. Nat Struct Biol.

[27] Denucci CC, Mitchell JS, Shimizu Y (2009). Integrin function in T-cell homing to lymphoid and nonlymphoid sites: getting there and staying there. Crit Rev Immunol.

[28] Berlin C, Bargatze RF, Campbell JJ, von Andrian UH, Szabo MC, Hasslen SR, Nelson RD, Berg EL, Erlandsen SL (1995). Butcher EC: alpha 4 integrins mediate lymphocyte attachment and rolling under physiologic flow. Cell.

[29] Chen J, Takagi J, Xie C, Xiao T, Luo BH, Springer TA (2004). The relative influence of metal ion binding sites in the I-like domain and the interface with the hybrid domain on rolling and firm adhesion by integrin alpha4beta7. J Biol Chem.

[30] Pan Y, Zhang K, Qi J, Yue J, Springer TA, Chen J (2010). Cation-pi interaction regulates ligand-binding affinity and signaling of integrin alpha4beta7. Proc Natl Acad Sci U S A.

[31] Bunting M, Bernstein KE, Greer JM, Capecchi MR, Thomas KR (1999). Targeting genes for self-excision in the germ line. Genes Dev.

[32] Alon R, Feigelson SW (2012). Chemokine-triggered leukocyte arrest: force-regulated bi-directional integrin activation in quantal adhesive contacts. Curr Opin Cell Biol.

[33] Wurbel MA, McIntire MG, Dwyer P, Fiebiger E (2011). CCL25/CCR9 interactions regulate large intestinal inflammation in a murine model of acute colitis. PLoS One.

[34] Higgins JM, Cernadas M, Tan K, Irie A, Wang J, Takada Y, Brenner MB (2000). The role of alpha and beta chains in ligand recognition by beta 7 integrins. J Biol Chem.

[35] Schon MP, Arya A, Murphy EA, Adams CM, Strauch UG, Agace WW, Marsal J, Donohue JP, Her H, Beier DR (1999). Mucosal T lymphocyte numbers are selectively reduced in integrin alpha E (CD103)-deficient mice. J Immunol.

[36] Mora JR, Bono MR, Manjunath N, Weninger W, Cavanagh LL, Rosemblatt M, von Andrian UH (2003). Selective imprinting of gut-homing T cells by Peyer’s patch dendritic cells. Nature.

[37] Izcue A, Coombes JL, Powrie F (2006). Regulatory T cells suppress systemic and mucosal immune activation to control intestinal inflammation. Immunol Rev.

[38] Geremia A, Biancheri P, Allan P, Corazza GR, Di Sabatino A (2014). Innate and adaptive immunity in inflammatory bowel disease. Autoimmun Rev.

[39] Ramirez-Carrozzi V, Sambandam A, Luis E, Lin ZG, Jeet S, Lesch J, Hackney J, Kim J, Zhou MJ, Lai J (2011). IL-17C regulates the innate immune function of epithelial cells in an autocrine manner. Nat Immunol.

[40] Smith PD, Ochsenbauer-Jambor C, Smythies LE (2005). Intestinal macrophages: unique effector cells of the innate immune system. Immunol Rev.

[41] Perse M, Cerar A (2012). Dextran sodium sulphate colitis mouse model: traps and tricks. J Biomed Biotechnol.

[42] Kim GD, Oh J, Park HJ, Bae K, Lee SK (2013). Magnolol inhibits angiogenesis by regulating ROS-mediated apoptosis and the PI3K/AKT/mTOR signaling pathway in mES/EB-derived endothelial-like cells. Int J Oncol.

[43] Choi JS, Kim KH, Lau LF (2015). The matricellular protein CCN1 promotes mucosal healing in murine colitis through IL-6. Mucosal Immunol.

[44] Sroor HM, Hassan AM, Zenz G, Valadez-Cosmes P, Farzi A, Holzer P, El-Sharif A, Gomaa FAM, Kargl J, Reichmann F (2019). Experimental colitis reduces microglial cell activation in the mouse brain without affecting microglial cell numbers. Sci Rep.

[45] Stefanich EG, Danilenko DM, Wang H, O'Byrne S, Erickson R, Gelzleichter T, Hiraragi H, Chiu H, Ivelja S, Jeet S (2011). A humanized monoclonal antibody targeting the beta7 integrin selectively blocks intestinal homing of T lymphocytes. Br J Pharmacol.

[46] Wang J, Shiratori I, Uehori J, Ikawa M, Arase H (2013). Neutrophil infiltration during inflammation is regulated by PILR alpha via modulation of integrin activation. Nat Immunol.

[47] Yago T, Petrich BG, Zhang N, Liu Z, Shao B, Ginsberg MH, McEver RP (2015). Blocking neutrophil integrin activation prevents ischemia-reperfusion injury. J Exp Med.

[48] Ley K, Laudanna C, Cybulsky MI, Nourshargh S (2007). Getting to the site of inflammation: the leukocyte adhesion cascade updated. Nat Rev Immunol.

[49] Cepek KL, Parker CM, Madara JL, Brenner MB (1993). Integrin-alpha-E-Beta-7 mediates adhesion of T-lymphocytes to epithelial-cells. J Immunol.

[50] Austrup F, Rebstock S, Kilshaw PJ, Hamann A (1995). Transforming growth factor-beta 1-induced expression of the mucosa-related integrin alpha E on lymphocytes is not associated with mucosa-specific homing. Eur J Immunol.

[51] Lefrancois L, Parker CM, Olson S, Muller W, Wagner N, Schon MP, Puddington L (1999). The role of beta7 integrins in CD8 T cell trafficking during an antiviral immune response. J Exp Med.

[52] Kotze PG, Ma C, Almutairdi A, Al-Darmaki A, Devlin SM, Kaplan G, Seow CH, Novak KL, Lu C, Ferraz JGP (2018). Real-world clinical, endoscopic and radiographic efficacy of vedolizumab for the treatment of inflammatory bowel disease. Aliment Pharm Ther.

[53] Tang MT, Keir ME, Erickson R, Stefanich EG, Fuh FK, Ramirez-Montagut T, McBride JM, Danilenko DM (2018). Review article: nonclinical and clinical pharmacology, pharmacokinetics and pharmacodynamics of etrolizumab, an anti-beta 7 integrin therapy for inflammatory bowel disease. Aliment Pharm Ther.

[54] Andersson EC, Christensen JP, Marker O, Thomsen AR (1994). Changes in cell adhesion molecule expression on T cells associated with systemic virus infection. J Immunol.

[55] Gabor MJ, Sedgwick JD, Lemckert FA, Godfrey DI, Korner H (2001). Lymphotoxin controls alpha(E) beta 7-integrin expression by peripheral CD8(+) T cells. Immunol Cell Biol.

[56] Weigmann B, Tubbe I, Seidel D, Nicolaev A, Becker C, Neurath MF (2007). Isolation and subsequent analysis of murine lamina propria mononuclear cells from colonic tissue. Nat Protoc.

[57] Sun H, Liu J, Zheng YJ, Pan YD, Zhang K, Chen JF (2014). Distinct chemokine signaling regulates integrin ligand specificity to dictate tissue-specific lymphocyte homing. Dev Cell.

[58] Maxwell JR, Brown WA, Smith CL, Byrne FR, Viney JL: Methods of inducing inflammatory bowel disease in mice. Curr Protoc Pharmacol 2009, Chapter 5:Unit5 58. 10.1002/0471141755.ph0558s47 22294404

[59] Laroui H, Ingersoll SA, Liu HC, Baker MT, Ayyadurai S, Charania MA, Laroui F, Yan YT, Sitaraman SV, Merlin D. Dextran sodium sulfate (DSS) induces colitis in mice by forming nano-lipocomplexes with medium-chain-length fatty acids in the colon. PLoS One. 2012;7(3):e32084. 10.1371/journal.pone.0032084 PMC3302894 22427817

