# Supplementary material for: A mutation that blocks integrin α4β7 activation prevents adaptive immune-mediated colitis without increasing susceptibility to innate colitis
Source: BMC Biol. 2020 Jun 10;18:64. doi: 10.1186/s12915-020-00784-6 (PMC7288534; doi:10.1186/s12915-020-00784-6)
Supplement: Supplementary file 1 — Additional file 1: Figure S1. The secondary lymphoid tissues other than GALT appear normal in β7-F185A KI and β7-KO mice. Representative histological sections of the peripheral lymph node (PLN), mesenteric lymph node (MLN) and spleen (SP) of WT, β7-F185A KI (KI) and β7-KO (KO) mice were analyzed by hematoxylin and eosin staining. Scale bars, 500μm. Figure S2. Expression of integrins β7 in splenic lymphocytes of WT and β7-F185A mice. (A) Quantitative PCR analysis of integrin β7 expression in splenic lymphocytes from WT and KI mice. Results are normalized to GAPDH. (B) Total (cell surface plus intracellular) protein expression of integrin β7 in splenic lymphocytes was determined by flow cytometry using permeabilized cells. Data are mean ± s.d. of at least 3 independent experiments (A-B). Figure S3. Impaired adhesion and transmigration of Jurkat T-β7 F185A cells. (A) Expession of β7 and CCR9 in Jurkat T-β7 WT and Jurkat T-β7 F185A cell lines were determined by flow cytometry. (B) Adhesion of Jurkat T-β7 WT, Jurkat T-β7 F185A and Jurkat T cells to MAdCAM-1 substrates at 1 dyn/cm2 or 2 dyn/cm2 before and after chemokine stimulation. (C) Transmigration of Jurkat T-β7 WT, Jurkat T-F185A and Jurkat T cells through MAdCAM-1-coated insert. Data are mean ± s.d. of at least 3 independent experiments (B C). *** P < 0.001; ns, not significant (Student’s t-test). Asterisk in B indicates the changes of total adherent cells. Figure S4. β7-F185A mutation does not affect αEβ7-mediated cell adhesion to E-cadherin substrates. (A) Expession of β7 and αE in Jurkat T-αEβ7 WT and Jurkat T-αEβ7 F185A cell lines were determined by flow cytometry. The numbers within the panels show the specific mean fluorescence intensities of FIB504 (anti-β7) and M290 (anti-αE) mAbs. (B) Adhesion of Jurkat T-αEβ7 WT, Jurkat T-αEβ7 F185A and Jurkat T cells to the immobilized E-cadherin substrates (40 μg/ml) at 1dyn/cm2 before and after chemokine stimulation. αEβ7-E-cadherin binding was inhibited by pre-treatme [file 12915_2020_784_MOESM1_ESM.pdf]

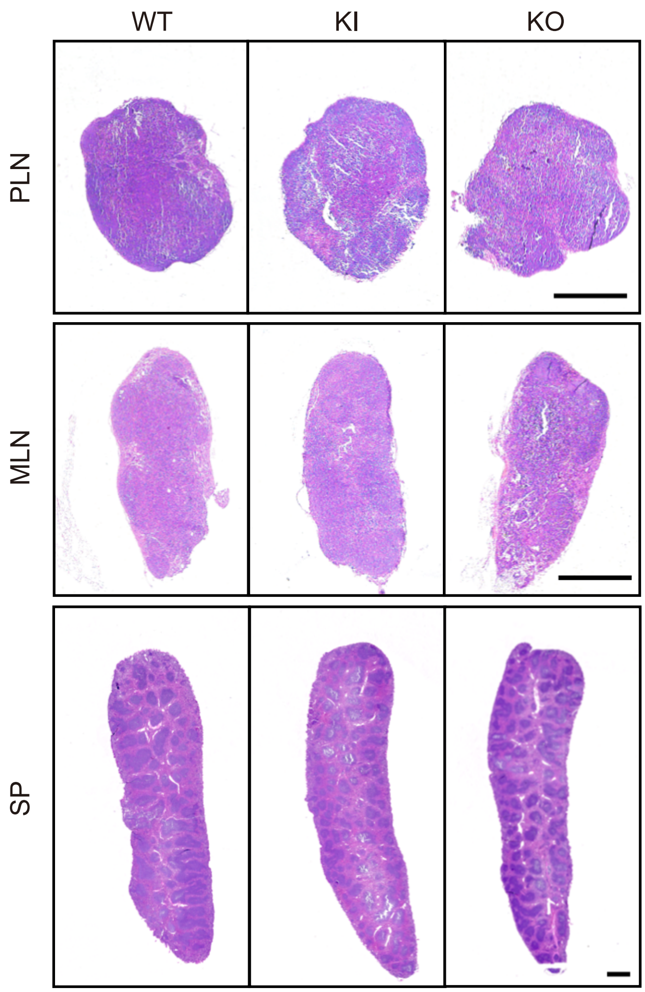

Figure S1. The secondary lymphoid tissues other than GALT appear normal in β7-F185A KI and β7-KO mice. Representative histological sections of the peripheral lymph node (PLN), mesenteric lymph node (MLN) and spleen (SP) of WT, β7-F185A KI (KI) and β7-KO (KO) mice were analyzed by hematoxylin and eosin staining. Scale bars, 500μm.

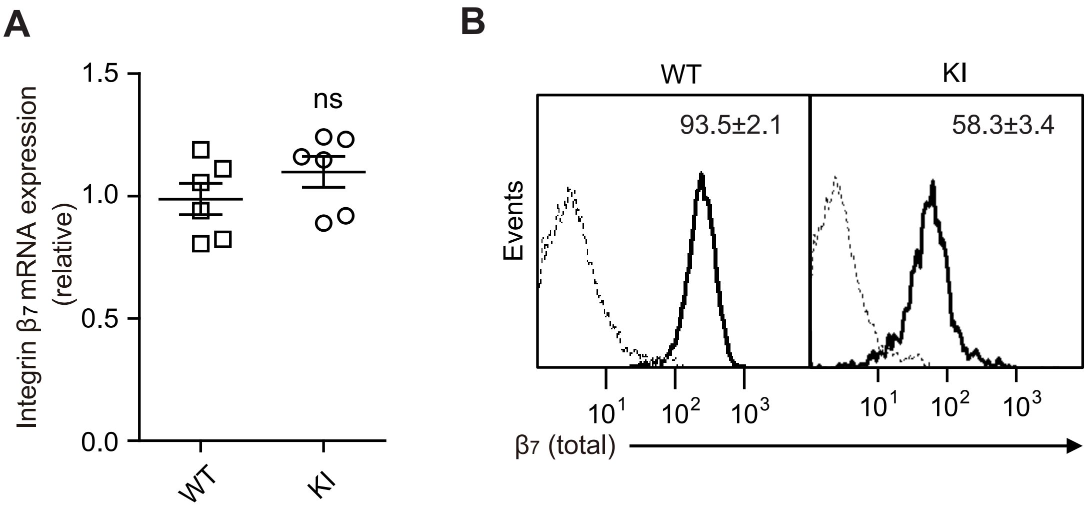

Figure S2. Expression of integrins β7 in splenic lymphocytes of WT and β7-F185A mice. (**A**) Quantitative PCR analysis of integrin β7 expression in splenic lymphocytes from WT and KI mice. Results are normalized to *GAPDH*. (**B**) Total (cell surface plus intracellular) protein expression of integrin β7 in splenic lymphocytes was determined by flow cytometry using permeabilized cells. Data are mean ± s.d. of at least 3 independent experiments (**A-B**).

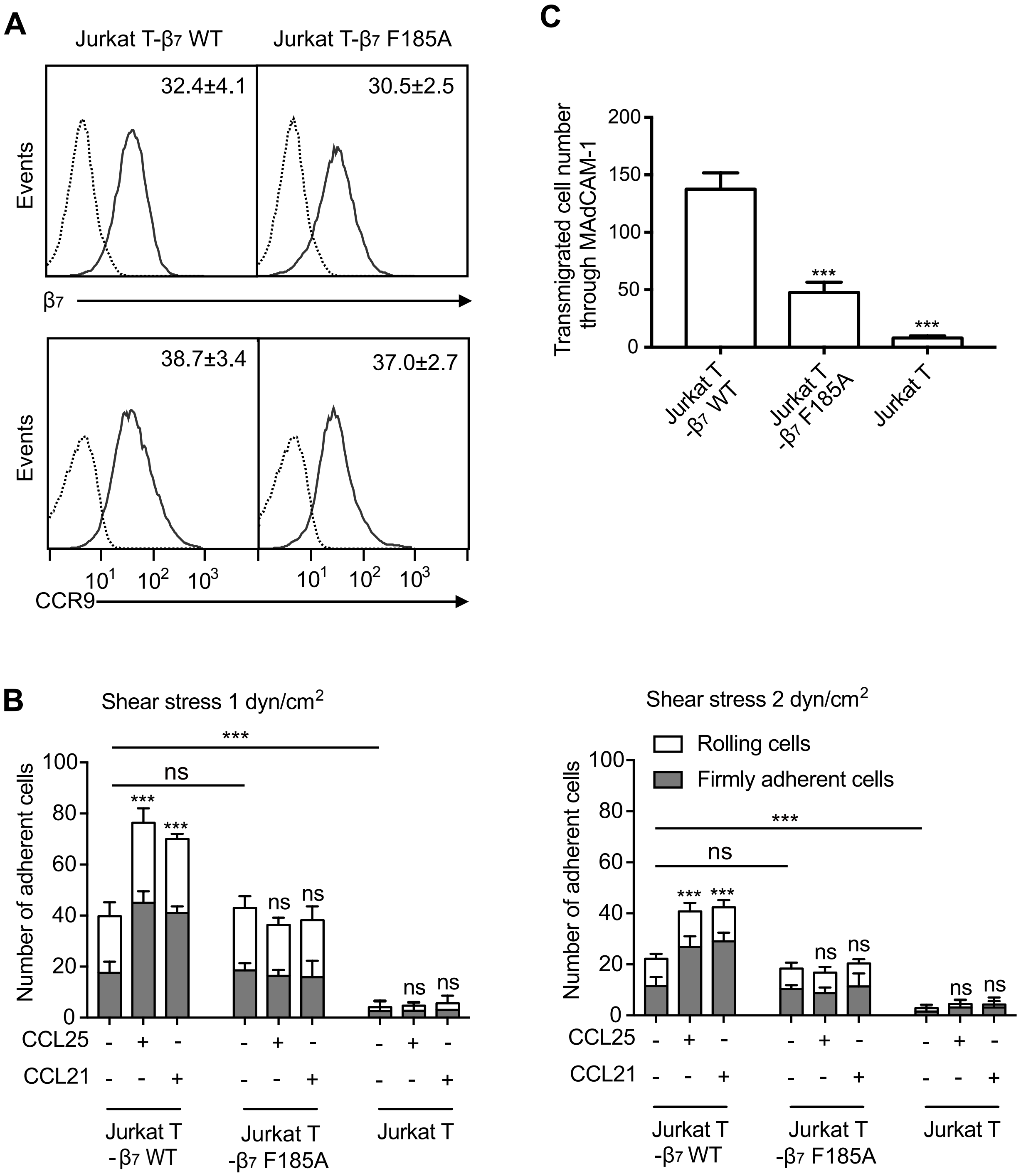

Figure S3. Impaired adhesion and transmigration of Jurkat T-β7 F185A cells. (**A**) Expession of β7 and CCR9 in Jurkat T-β7 WT and Jurkat T-β7 F185A cell lines were determined by flow cytometry. (**B**) Adhesion of Jurkat T-β7 WT, Jurkat T-β7 F185A and Jurkat T cells to MAdCAM-1 substrates at 1 dyn/cm² or 2 dyn/cm² before and after chemokine stimulation. (**C**) Transmigration of Jurkat T-β7 WT, Jurkat T-F185A and Jurkat T cells through MAdCAM-1-coated insert. Data are mean ± s.d. of at least 3 independent experiments (**B**-**C**). *** *P* < 0.001; ns, not significant (Student's *t*-test). Asterisk in **B** indicates the changes of total adherent cells.

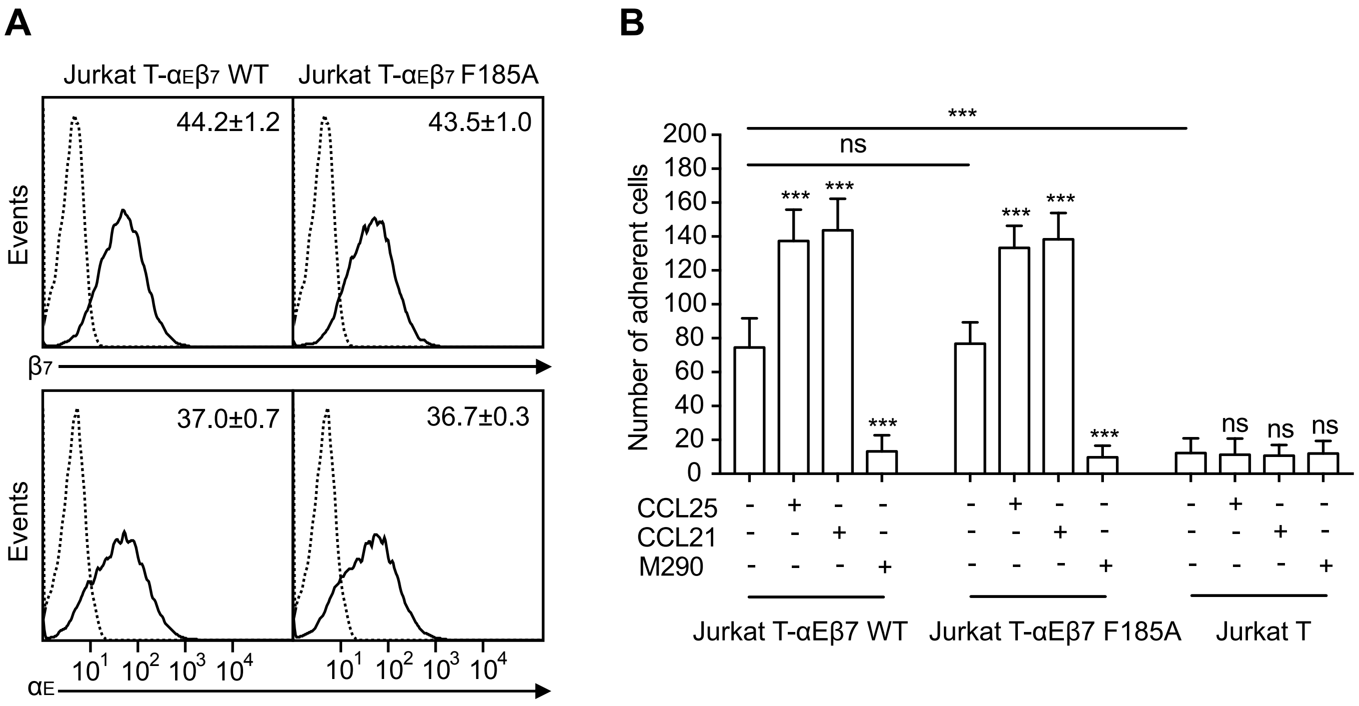

Figure S4. β7-F185A mutation does not affect αEβ7-mediated cell adhesion to E-cadherin substrates. (**A**) Expession of β7 and αE in Jurkat T-αEβ7 WT and Jurkat T-αEβ7 F185A cell lines were determined by flow cytometry. The numbers within the panels show the specific mean fluorescence intensities of FIB504 (anti-β7) and M290 (anti-αE) mAbs. (**B**) Adhesion of Jurkat T-αEβ7 WT, Jurkat T-αEβ7 F185A and Jurkat T cells to the immobilized E-cadherin substrates (40 μg/ml) at 1dyn/cm^2 before and after chemokine stimulation. αEβ7-E-cadherin binding was inhibited by pre-treatment of cells with 10 μg/ml αE blocking antibody M290. Data are mean ± s.d. of at least 3 independent experiments (**A**- **B**).*** $P < 0.001$; ns, not significant (Student's *t*-test).

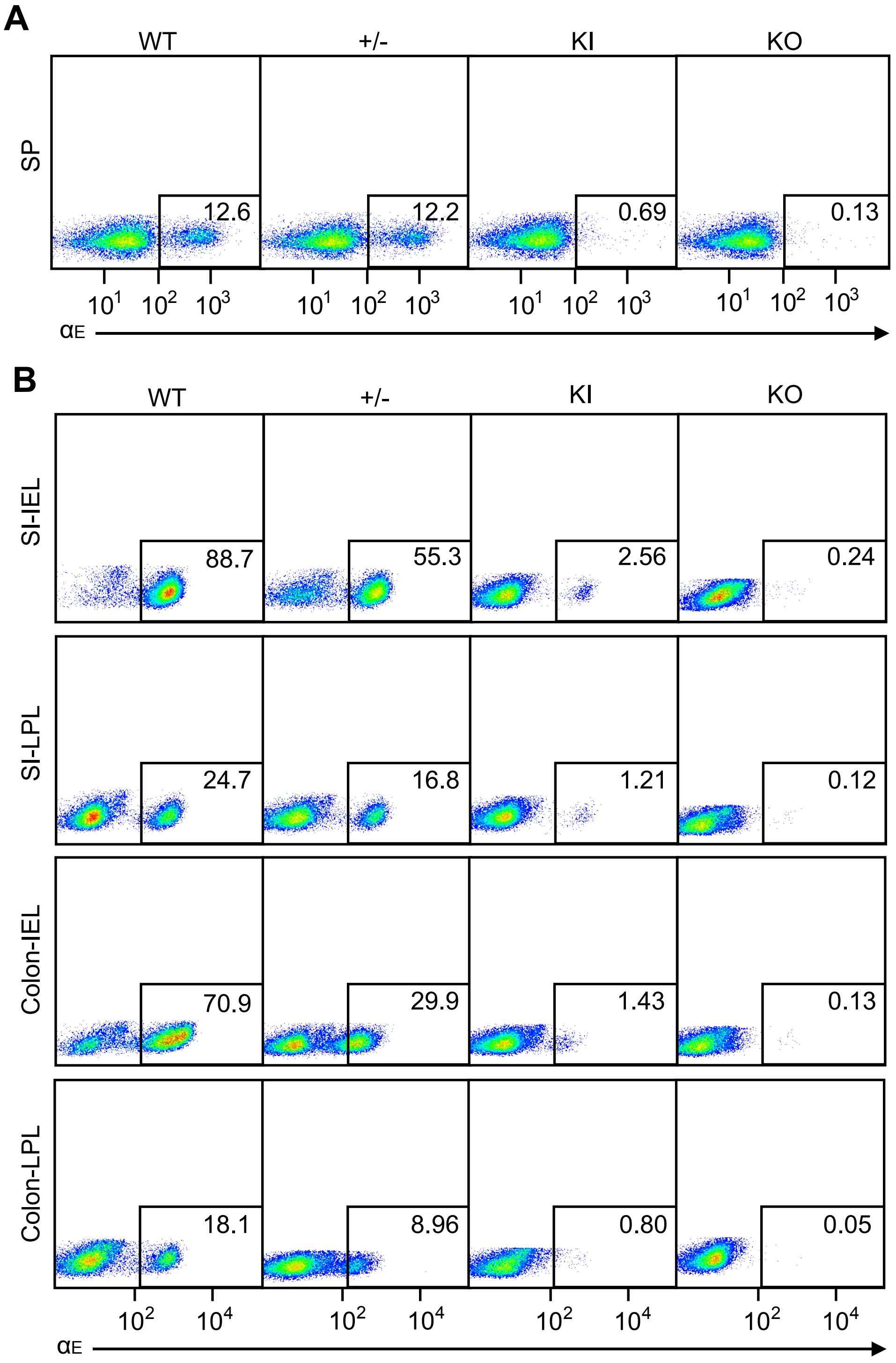

C

Genotype	WT	+/-	KI	KO
SI-IEL α_E (MFI)	333.7±40.7	303.5±30.8	192.5±27.8^{AA}	140.0±24.8^{AAA}
SI-LPL α_E (MFI)	307.3±21.4	274.0±51.1	151.7±38.5^{AA}	139.1±10.9^{AAA}
Colon-IEL α_E (MFI)	304.4±58.2	269.4±43.6	146.7±24.7^{AA}	131.0±13.6^{AAA}
Colon-LPL α_E (MFI)	300.3±33.4	254.7±54.4	148.7±27.1^{AA}	128.1±15.9^{AAA}

Figure S5. Integrin α_E^+ lymphocytes in spleen, SI and colon. (**A**) Expression of α_E in WT, +/-, KI and KO splenic lymphocytes was determined by flow cytometry. (**B**-**C**) Expression of α_E in intestinal IEL and LPL was detected by flow cytometry. The numbers within the panels (**A**-**B**) show the percentage of α_E^+ lymphocytes. The numbers within the table (**C**) show the specific mean fluorescence intensities of M290 (anti-α_E) mAb in α_E^+ lymphocytes. Data are mean ± s.d. of at least 3 independent experiments (**A**-**C**). $^{AA}P < 0.05$; $^{AAA}P < 0.001$ (Student's *t*-test in **C**). SP, spleen; SI, small intestine; IEL, intraepithelial lymphocyte; LPL, lamina propria lymphocyte.
