# Supplementary material for: A mutation that blocks integrin α4β7 activation prevents adaptive immune-mediated colitis without increasing susceptibility to innate colitis
Source: BMC Biol. 2020 Jun 10;18:64. doi: 10.1186/s12915-020-00784-6 (PMC7288534; doi:10.1186/s12915-020-00784-6)
Supplement: Supplementary file 2 — Additional file 2: Table S1. The analysis of integrin β7-high splenic lymphocytes subsets in WT mice. Table S2. The list of primers used for genotyping. Table S3. The list of primers used for real-time quantitative PCR analyses [file 12915_2020_784_MOESM2_ESM.pdf]

Table S1. The analysis of integrin β7-high splenic lymphocytes subsets in WT mice

**$CD19^+$ B cells**	**$CD3^+$ T cells**	**$CD8^+$ T cells**	**$CD4^+$ T cells**	**$CD4^+$ $CD25^+$ Treg cells**
3.90%±0.63%	91.13%±2.08%	79.05%±2.87%	8.74%±0.72%	1.42%±0.28%

Integrin β7-high splenic lymphocytes were stained with CD3, CD19, CD4, CD8 and CD25 mAbs. The percentages of $CD19^+$ B cells, $CD3^+$ T cells, $CD8^+$ T cells, $CD4^+$ T cells and $CD4^+$ $CD25^+$ Treg cells were shown in the Table. Data are mean ± s.d. (n=4 mice).

Table S2. The list of primers used for genotyping

Gene	Forward sequence (5' to 3')	Reverse sequence (5' to 3')	Species
Itgb7	TCTACAGGAGGACCAACTGAG	GATGTGCTGCAAGGCGATTAAG	mouse

Table S3. The list of primers used for real-time quantitative PCR analyses

Gene	Forward sequence (5' to 3')	Reverse sequence (5' to 3')	Species
IL-6	CTGCAAGAGACTTCCATCCAGTT	GAAGTAGGGAAGGCCGTGG	mouse
TNF-α	AGTGACAAGCCTGTAGCCC	GAGGTTGACTTTCTCCTGGTAT	mouse
IL-1β	CGGCACACCCACCCTG	AAACCGCTTTTCCATCTTCTTCT	mouse
Itgb7	GCAGTACAGGGATTGTGCAGA	GGTTGTCTATTGTCCTTTCTTTGC	mouse
GAPDH	CGGAGTCAACGGATTTGGTC	GACAAGCTTCCCGTTCTCAG	mouse
